# The Zika epidemic and abortion in Latin America: a scoping review

**DOI:** 10.1186/s41256-018-0069-8

**Published:** 2018-05-03

**Authors:** Mabel Carabali, Nichole Austin, Nicholas B. King, Jay S. Kaufman

**Affiliations:** 1 Department of Epidemiology, Biostatistics and Occupational Health, Faculty of Medicine, McGill University, 1020 Pine Avenue West, Purvis Hall Room 17A, Montreal, QC H3A 1A2 Canada; 2 Biomedical Ethics Unit, McGill University, Montreal, QC Canada

**Keywords:** Zika, Congenital Zika syndrome, Latin America, Abortion, Public health practice, Maternal and child health

## Abstract

**Background:**

Latin America presently has the world’s highest burden of Zika virus, but there are unexplained differences in national rates of congenital malformations collectively referred to as Congenital Zika Syndrome (CZS) in the region. While Zika virulence and case detection likely contribute to these differences, policy-related factors, including access to abortion, may play important roles. Our goal was to assess perspectives on, and access to, abortion in Latin America in the context of the Zika epidemic.

**Methods:**

We conducted a scoping review of peer-reviewed and gray literature published between January 2015 and December 2016, written in English, Spanish, Portuguese, or French. We searched PubMed, Scielo, and Google Scholar for literature on Zika and/or CZS and abortion, and used automated and manual review methods to synthesize the existing information.

**Results:**

36 publications met our inclusion criteria, the majority of which were qualitative. Publications were generally in favor of increased access to safe abortion as a policy-level response for mitigating the impact of CZS, but issues with implementation were cited as the main challenge. Aside from the reform of abortion regulation in Colombia, we did not find evidence that the Zika epidemic had triggered shifts in abortion policy in other countries.

**Conclusion:**

Abortion policy in the region remained largely unchanged following the Zika epidemic. Further empirical research on abortion access and differential rates of CZS across Latin American countries is required.

**Electronic supplementary material:**

The online version of this article (10.1186/s41256-018-0069-8) contains supplementary material, which is available to authorized users.

## Background

Zika virus is a vector borne disease primarily transmitted to humans by mosquitoes or via sexual transmission [1–5]. The illness itself tends to be mild and self-limited [3–6], but diagnosis is challenging: no rapid diagnostic tests are available, and given the insufficient specificity of serological tests, specialized molecular diagnostic analyses (i.e., RT-PCR) are required to differentiate Zika from other flaviviruses transmitted by the same vector [1, 4, 6–8]. Vaccines are not yet available, and vector control (together with prevention of sexual transmission) is presently the main public health action to address the spread of disease [7–9].

Although Zika had not previously been associated with severe forms of disease [9, 10], it has recently been linked to several adverse outcomes [3–5, 8]. Zika is now associated with a rise in the incidence of Guillain-Barré Syndrome (GBS), a type of flaccid ascending paralysis usually triggered by infections [7, 11, 12]. More alarmingly, Zika has recently been associated with adverse reproductive outcomes including miscarriage, fetal demise, stillbirth, and congenital malformations such as microcephaly (with or without severe brain alterations such as holoproscencephaly, agyria, or anencephaly), intrauterine growth restriction, and ocular abnormalities. These fetal anomalies are now collectively referred to as Congenital Zika Syndrome (CZS) [13–19].

The incidence of CZS in pregnancies of Zika-infected women is estimated to be between 1 and 13%, but possibly as high as 42% [19–23]. Diagnosis of CZS, much like diagnosis of Zika, is complex and involves the investigation of a number of factors, including symptoms, residence in or travel to Zika endemic zones, a positive Zika diagnostic test, and presence of any of the aforementioned fetal malformations [7, 13, 24]. CZS includes a wide range of malformations that range from mild to life-threatening: infants born with CZS may require lifelong assistance and access to quality health services, and their families may require psychological, social, and economic support [16, 24–26].

### Public health implications

Beginning in late 2015, the world witnessed the emergence of Zika in the Americas and its re-emergence in other regions [4, 14, 15, 27]. While researchers are working to better understand the causal link between Zika and the conditions constituting CZS, observational evidence and evidence from animal models prompted the World Health Organization (WHO) to declare Zika a Public Health Emergency of International Concern (PHEIC) in February 2016 [28]. Aside from emphasizing the need for accurate identification of Zika-related malformations, the statement also encouraged health authorities to educate pregnant women and women in their childbearing years about their risks, and to follow and counsel women already exposed to Zika [7, 24, 28]. Zika infection can be asymptomatic and access to accurate diagnostic tests is often limited, so many countries began to promote antenatal screening for Zika and CZS [7, 29, 30]. Some countries advised women to delay pregnancies altogether and/or encouraged the use of family planning measures to reduce the risk of undesirable birth outcomes [29–32].

By February 1st, 2017, one year after the PHEIC was declared, 76 countries had reported the presence of Zika; 70 of these countries only began to detect and report cases in 2015, and 59 have documented outbreaks. Of the 205,013 cumulative confirmed cases of Zika infection across the world, 130,840 were in Brazil and 9799 were in Colombia [33]. In addition, 2656 cases of CZS had been reported worldwide, with Brazil accounting for the majority of these cases (89%, *n* = 2366), followed by Colombia (3.2%) and the Dominican Republic (0.8%); placing the highest burden of Zika and confirmed CZS in the Latin American region [33].

### Differential distribution of disease

There are likely many reasons for the variation in the number of CZS cases from country to country. While national differences in disease surveillance and reporting may explain some of this trend, various micro and macro-level factors such as national/regional differences in the force of Zika infection, the number of susceptible individuals, exposure and transmission patterns, environmental and contextual considerations related to ethnicity, and the Zika strain itself could contribute to observed national-level differences [3, 5, 15, 18, 27, 34, 35]. Access to and utilization of family planning services, as well as behavioral changes in response to public health initiatives, may also influence the observed variation [26, 36, 37]. Finally, policy-related factors – in particular, access to abortion – may play a role in determining national rates of CZS [21, 38–40], as access to abortion would afford the option of (legal) termination to women with Zika-affected pregnancies. However, even in the absence of legal abortion, there may still be important regional differences in the use of self-induced or *un*regulated abortion practices, which could also have an impact on national rates of CZS.

### Abortion in Latin America

Abortion is illegal in most Latin American countries, and exceptions (where they exist) are often only considered under very limited circumstances, such as in the presence of specific fetal malformations [37, 41–47]. Abortion is legal and largely unrestricted in only a handful of Latin American countries (Cuba, Uruguay, and Puerto Rico) [48]; first trimester abortion is also legal in Mexico City, although it remains illegal in other parts of the country. However, regardless of the legality of abortion, local social norms, socio-economic factors, and religious values may impact women’s likelihood of accessing abortion services [42, 47, 49]. Although reproductive health policy the in the region is largely restrictive, policy nuances, abortion practices, and Zika/CZS incidences are fairly heterogeneous (Additional file 1) [7, 44, 45, 48, 50–52].

While the moral/ethical debate over abortion access is certainly not new or unique to Latin America, other public health crises like Rubella and HIV/AIDS have historically shaped reproductive health policy in the region. It is reasonable to posit that the Zika epidemic may play a similar role [29, 53, 54]. However, the extent to which women and countries have used abortion as a way of managing Zika-related risks remains unclear given the recency of the epidemic, and the question of whether abortion (regardless of legality) is viewed as morally acceptable at the personal and/or community level as a strategy for CZS management is complex and difficult to assess. These issues are compounded by a lack of high-quality, national-level abortion data, as well as a lack of data on the occurrence of self-induced or unregulated abortion; both of these limitations currently make challenging (or impossible, in some settings) to quantify the regional use of abortion as a response to Zika and CZS, subsequently limiting the amount of original, quantitative research available for synthesis. In light of these challenges, we conducted a scoping review (in lieu of a more traditional systematic review) to survey the available literature on Zika, CZS, and abortion in Latin America. Our primary goal was to investigate policies and discourse regarding abortion in the context of the Zika epidemic, particularly as an option for managing the burden of CZS and its component conditions.

## Methods

Given the timing of the epidemic, we anticipated that the literature on abortion and Zika in Latin America would be limited, recent, and diverse. In the interest of capturing as much relevant literature as possible, we conducted a scoping review, which is a type of review used to explore a broad and often complex range of evidence from various sources. While both systematic and scoping reviews rely on a rigorous and clearly-defined search strategy, scoping reviews generally offer more flexibility with respect to inclusion criteria and evidence synthesis. Scoping reviews are particularly useful in identifying research gaps and informing future practice [55–57].

### Search strategy and data extraction

We searched three electronic databases (PubMed, Scielo, and Google Scholar) for peer-reviewed and grey literature using a combination of both MeSH (Medical Subject Headings) and free-text forms of the terms listed in Additional file 2. The inclusion of grey literature was important in this case due to the relative novelty of the Zika outbreak and the possibility that relevant publications might not appear in conventional search engines. We did not impose restrictions on study or document type: we were primarily interested in original, quantitative research, but because abortion access is likely influenced by both national mandates and sociocultural norms, we also opted to include qualitative accounts and publications on the bioethical arguments for modifying abortion regulations in response to the Zika epidemic. Documents were eligible for inclusion if they were written in English, Spanish, Portuguese or French, published (or e-published) from January 2015 to December 2016, and contained the report, description, or analysis of abortion and Zika or CZS-related cases in humans in Latin America. As the formal definition of CZS was a relatively recent phenomenon, we included more general search terms (microcephaly, birth defects) in addition to “congenital Zika syndrome” to ensure that our search captured as much pertinent literature as possible. We excluded documents reporting abortion related to Zika or CZS in other countries or regions.

All references retrieved through the initial search were saved in an EndNote® library and reviewed for relevance. Additional publications were identified by reviewing the reference lists of relevant papers. We created a data extraction form to collect data on the general content and quality of retrieved publications [58]. We used this form to capture detailed information on six a priori elements, indicating whether the selected publications i) reported or identified actual cases of Zika-related malformations; ii) discussed any type of Zika-related malformations (microcephaly only, ocular malformations only, or any CZS) iii) considered postponing pregnancy to be a viable or effective preventive strategy against Zika-related complications; iv) considered abortion to be a viable or effective option in response to the Zika epidemic; v) discussed or advocated for changes in healthcare systems, including improved surveillance, antenatal care/screening, and family planning programs; and vi) discussed or advocated for changes in abortion policy in response to the Zika epidemic (Additional file 3).

### Data synthesis and analysis

In addition to the aforementioned manual review process, publications meeting our eligibility criteria were analyzed using QDA Miner (Provalis Research, Montreal, Canada), software designed to facilitate both quantitative and qualitative (content) analysis. We conducted a content analysis of all relevant publications, treating each document as a “case”. Information was descriptively synthetized using the SIMSTAT feature of the software, in which the frequency of key words such as “abortion”, “ethics”, and “policy” was listed by case and among all reviewed cases. We conducted a latent content analysis using the WORDSTAT feature of the software to assess the contextual meaning of these key words. All cases were also reviewed and assessed by two investigators (MC, NA). The review was compiled in accordance with PRISMA guidelines (Additional file 4).

## Results

**Fig. 1:**
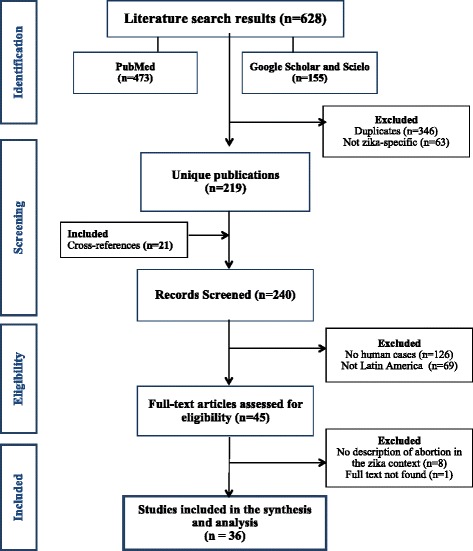
Data search and synthesis flowchart of reviewed literature about abortion, Zika, and Congenital Zika Syndrome (CZS) in Latin America

Our initial search strategy returned 628 documents, of which 36 were retained for analysis (Fig. 1). Only two of the publications were original, quantitative studies; the majority (93%) were letters to the editor, editorials, and commentaries. 20 publications discussed the Latin American region as a whole; the remaining 16 assessed a single country or a small number of countries (most commonly Brazil, El Salvador, and Colombia, in order of frequency).

Key themes emerging from this search can be loosely partitioned into two broad categories: i) abortion policy and abortion decision-making, and ii) health care systems and practitioners. Ethical arguments were common across both of these overarching themes: most documents (86%) called for modifications to national or regional abortion-related legislation in light of the Zika epidemic, often framing this as a human rights issue and emphasizing concerns about women’s agency and gender equity in Latin America (Table 1).

**Table 1:** Descriptive characteristics of the documents and content information about abortion and Zika

Content information	*n* (%)
Discussed ethical considerations about Zika, CZS and abortion	20 (74)
Considered postponing pregnancy to be a viable option to decrease the burden of CZS	7 (19)
Considered abortion to be a viable option to decrease the burden of CZS	31 (86)
Advocated for changes in Zika surveillance systems	8 (22)
Advocated for changes in antenatal screening/care for CZS	9 (25)
Discussed family planning/contraception options (aside from pregnancy delay) for CZS management	26 (72)
Explicitly discussed and/or advocated for changes in abortion policy due to Zika/CZS	31 (86)

### Abortion policy and abortion decision-making

While abortion is routinely provided as a health care service in some Latin American settings (for example, Cuba and Uruguay), it is completely illegal in three of the countries with documented cases of Zika and/or CZS (Dominican Republic, Nicaragua and El Salvador), and women who seek abortion may face considerable penalties. In El Salvador, where the government formally recognizes a fertilized egg as a person, suspected violations of the abortion restriction typically result in imprisonment and homicide charges, and prison sentences can range from 30 to 50 years for pregnancies over 20 weeks [49, 59]. Other countries, such as Colombia and Brazil, have decriminalized abortion only under exceptional circumstances [41, 45, 49–52, 59–63]. In Brazil, abortion is decriminalized in cases of rape or incest, conditions that threaten the life of the woman, and severe fetal brain malformations (i.e. anencephaly) [41, 45, 52]. However, although Zika may cause anencephaly, abortion due to Zika/CZS alone is not currently legal. Despite increased efforts on the part of Brazilian abortion rights advocates in response to the Zika epidemic, the only law concerning abortion and Zika in Brazil as of late 2016 was proposal PL 4396/2016, which would increase abortion-related incarceration time for women who terminate pregnancies due to CZS [41, 52]. Colombia, which decriminalized abortion under specific circumstances in 2006, is slightly more progressive: in addition to advising women to delay their pregnancies in due to the Zika outbreak, the Colombian Ministry of Health issued a resolution allowing abortion after a conclusive diagnosis of Zika/CZS [30, 39, 45, 59].

While national-level abortion policies remain quite restrictive, individual-level abortion decisions may not always align with local regulations. This is true across most settings, but the divergence between local policy and individual behavior may be particularly notable in the context of the Zika epidemic. One study in our review reported a higher than expected demand for abortion medications (via channels outside of the formal healthcare system) in most of the Latin American countries that had issued pregnancy-related advisories in response to the Zika epidemic (50). Individual-level decision-making may also be heterogeneous across settings and personal circumstances: our review suggested that religion, education, socioeconomic status, and access to health care may play a role in the reproductive decisions of Zika-infected women, whether this decision concerns the termination or continuation of a current pregnancy, or the postponement of a future pregnancy [41, 45, 52, 59, 64, 65]. Some articles discussed the possibility that some women could use revised or relaxed abortion regulations as an excuse to abort pregnancies unaffected by Zika, but most were more focused on women’s use of abortion as a strategy for risk management, given the fear of Zika-related malformations [45, 50–52, 59–68]. Although abortion was framed by most (*n* = 31) of the retrieved articles as a reasonable option for Zika and CZS management, many articles noted that the procedure is often met with extensive and unconditional disapproval from the general population, decision makers, and health practitioners (we expand on this in the next section) [37, 49, 59, 61, 69]. Prevailing cultural and religious beliefs were often cited as a reason for this aversion [49, 59, 60, 65, 68–70].

Many of the documents returned by our search offered fairly extensive discussions of women’s autonomy and reproductive rights in the context of Zika/CZS. A common argument among publications in favor of expanded abortion access was that women have a right to their own bodies, and this self-ownership entitles them to make their own pregnancy decisions [45, 51, 52, 60, 65]. Although this point applies to all women, it may be particularly meaningful with respect to women of low socioeconomic position (SEP), who are more vulnerable to Zika and have limited access to information about family planning and contraceptives [37, 59, 64–66, 71]. Various authors also argued that access to safe and legal abortion would make women less likely to seek unsafe and potentially life-threatening procedures; others conceptualized abortion as a resource to avoid unnecessary pain or suffering for both pregnant women and children with severe malformations, particularly when the CZS-related malformations are so severe that there is a high risk of stillbirth or neonatal death [41, 45, 52, 62, 63]. However, while most of the publications in our review provided ethical justifications in favor of expanding abortion access in response to the Zika outbreak, some also acknowledged the potential adverse repercussions of expanded access: for example, since the absolute risk of malformation may be very low, and because many of the CZS anomalies may not be detectable at early gestational stages (when abortion is safest), increased abortion access may lead to termination decisions that are motivated by exaggerated risk perception instead of confirmation of true CZS [45, 71].

### Health care systems and practitioners

Many publications in our review noted health practitioners’ often limited or restricted ability to provide safe abortions to women who need them, and the generally limited access to family planning programs in much of Latin America, including the use of emergency contraceptives (i.e.: the “morning after” pill, using estrogen and/or progestin pills combined or alone, including Plan B® or other contraceptive pills) [37, 51, 52, 60, 63, 64, 66, 71, 72]. Others acknowledged that some practitioners may face moral dilemmas given the incongruence between abortion laws and their personal beliefs, and some may weigh individual women’s socioeconomic situations as a factor when deciding whether to provide services [41, 52, 67, 69]. Furthermore, practitioners may be dissuaded from providing abortions due to nebulous constraints on the situations in which abortion is legal for fear of losing their license [41, 45, 49]. These concerns, while not specific to the Zika epidemic, may become exacerbated if abortion demand is in fact increased in Zika-affected contexts. A number of publications also discussed the possibility of an increase in illegal or unsafe abortion (and a corresponding increase in maternal morbidity and mortality rates) in Zika-affected countries where abortion is illegal or in settings where socio-cultural factors dissuade women from openly seeking professional care [45, 49–52, 63].

Various preventive initiatives were also frequently discussed by the publications in our review. Many articles described the national pregnancy advisories currently in place in certain countries, but most did not view this type of initiative as particularly effective, at least in the absence of complementary services like improved access to, and uptake of, high-quality contraception in high-risk populations. A number of publications discussed the importance of improved antenatal screening in light of the Zika outbreak, as well as the use of existing policies and protocols implemented in previous public health responses to Rubella, HIV/AIDS, and other TORCH entities (toxoplasmosis, rubella, cytomegalovirus, herpes simplex, and HIV) to establish a framework for the management of Zika and related malformations [41, 53, 64, 68]. Although no articles in our review indicated that an increase in screening resulted in an increase in abortions, some authors mentioned that, given the possibility of early detection of fetal malformations, access to adequate antenatal care was important in providing women with information and options [37, 51, 64, 67, 71, 72].

Some publications offered utilitarian arguments about the cost-effectiveness of expanded family planning programs and abortion in Zika/CZS management [37, 45, 68, 73, 74], particularly when governments do not have the resources to adequately support the treatment and long-term management of children with disabilities. For example, the Brazilian government provides a three-year subsidy to mothers of children with any CZS malformation. However, such malformations tend to be permanent conditions, and (according to some authors) preventing these births may be less expensive for governments and for families than the lifetime cost of supporting individuals with chronic disabilities [37, 68, 74].

## Discussion

This review assessed a range of largely qualitative publications on Zika, CZS, and abortion in the Latin American context following the recent Zika outbreak. Our findings point to considerable ethical and pragmatic complexity at every level of this issue, from individual-level decisions of Latin American women following a Zika or CZS diagnosis, to national-level decisions about Zika prevention and abortion provision.

Several scholarly bodies have called for expanded access to safe abortion in Latin America, particularly given contemporary concerns about Zika and CZS [41, 45, 50–52, 61, 63, 75]. However, our findings suggest that, even where abortion is legal, social and religious norms may dissuade health care practitioners from providing abortion services, and/or prevent women from accessing services [45, 49–52, 63], which is supported by previous research about abortion worldwide [42, 76–79]. Although it did not emerge as a key theme in our review, it is contextually important to note that the Catholic church (and increasingly, Evangelical Protestantism) plays a key role in promoting restrictive abortion legislation in the region: religious leaders and institutions are uniquely positioned to influence the actions of politicians and policymakers, and they have historically exercised this power on matters concerning reproductive health policy [79–81]. While the political influence of religious institutions is likely a key barrier in abortion liberalization in Latin America, our review suggests that disdain for abortion is often shared by the general population in many countries [49, 63, 69]. Many of the publications in our review discussed the pervasive stigmatization of abortion in the Latin American context; these arguments are mirrored in a 2014 poll [82] in which a clear majority of respondents in nearly every Latin American country (with the notable exception of Uruguay and, to a lesser extent, Chile) reported strong opposition to abortion in any circumstance [83]. Likewise, another poll conducted in Brazil in February 2016 reported that 58% of women were against abortion access in the case of Zika infections, and 51% held the same position even in the presence of microcephaly [52, 59]. Nonetheless, despite a cultural climate of resistance toward increased abortion access in most of the region, recent descriptive evidence suggests an increase in demand (outside of the formal healthcare system) for abortion medications like mifepristone and misoprostol [50, 51, 83]. Many of the papers in our review also cautioned that, in the absence of legal and accessible abortion, women who receive a diagnosis of CZS may instead turn to unsafe and unregulated abortions, potentially putting their lives at risk [42–44, 47, 49–52, 61, 75].

Although the majority of documents in this review supported increased access to abortion, specifically in the context of Zika/CZS, their rationale often differed considerably. Some authors argued that the economic burden of supporting thousands of children with lifelong disabilities may outweigh the cost of creating access to safe abortion [37, 42, 68, 73, 74, 78]. For example, the estimated direct medical cost for a single case of microcephaly in Latin America is $91,102 USD per lifetime, even before considering additional out-of-pocket costs for childcare and home modifications [37, 74]. If women are turning to unsafe abortion (an important cause of maternal morbidity) following Zika diagnosis, treating the complications associated with these procedures is also costly: one study estimated an average cost of $94 USD per case (2006 dollars) in Latin America, and a regional health system cost of $274 million USD annually [84]. Provision of safe abortion may be less expensive than treating these complications, but this appears to be heavily dependent on the context and provider: for example, the cost of safe abortion in Colombia ranged from $45 USD in a specialty clinic to up to $213 USD in secondary/tertiary facilities, whereas the average cost per case of treating complications of unsafe abortion was $141 [85]. It is also important to consider that there may be intangible costs related to anxiety, psychological suffering, stigma, and fatigue derived from either carrying or terminating a pregnancy following a diagnosis of Zika or CZS [7, 17, 37, 42, 43, 47].

A few of the publications in our review discussed pragmatic considerations related to the design and implementation of new abortion services in countries aiming to expand abortion access, which is (again) relevant both within and beyond the context of the Zika epidemic. For instance, health care practitioners will require practical training, but our review suggests that they may also benefit from initiatives designed to destigmatize the practice of abortion. Counseling services would likely be advantageous for women, couples, and practitioners [37, 42, 47, 53, 66, 72, 77, 86, 87]. Publications also offered practical advice with respect to preventive initiatives like pregnancy delay advisories, antenatal screening, and contraception provision. Expansion of family planning programs and access to high-quality contraception may be particularly important in Latin American Zika/CZS management, given the current lack of access to abortion [42, 43, 47, 73, 75–78, 88].

### Limitations

In an effort to capture literature published at the height of the Zika epidemic, we restricted the timeframe of our search to material published in 2015–2016. However, this may be too narrow to detect any substantial changes from the beginning of the outbreak to the present. Furthermore, given the small number of original research articles (*n* = 2) yielded by our search strategy, we were unable to perform a formal quality assessment, assess publication bias, or conduct any quantitative synthesis of the available evidence. Although some documents contained valuable information on health inequalities and social determinants of health related to Zika, CZS, and abortion, the heterogeneity of these documents was another barrier to a more comprehensive synthesis.

The majority of the articles retrieved by our search strategy were in favor of women’s right to abortion; as such, there is a potential risk for overestimation of abortion demand in the Zika/CZS context. We are also concerned about differential Zika/CZS detection and reporting across Latin American countries given considerable differences in infrastructure, healthcare systems, and public health priorities. Finally, the documents retrieved by this review contained information only about the countries with the highest rates of CZS in Latin America; our findings may not be reflective of the entire region.

## Conclusions

Zika is a pressing public health concern in Latin America, but surprisingly little is currently known about the extent to which local policies influence Zika-related outcomes like CZS. Our findings, while generally supportive of increased access to safe abortion as a method of CZS management, highlight the ethical and practical complexity of expanding these services in Latin America due to sociopolitical, cultural, and religious beliefs. Findings were mainly qualitative: limitations on regional abortion data introduce a considerable challenge to understanding the extent to which women currently use abortion to manage CZS risks, but this remains an exceptionally important next step given regional differences in rates of CZS. Additional research is necessary to better understand the impact of reproductive health policy on Zika and CZS-related pregnancy outcomes in Latin America.

## Additional files

Additional file 1: Description of country-specific information on cumulative Zika cases, CZS cases, abortion regulations, and abortion demand in 13 Latin American countries. (PDF 30 kb)

Additional file 2: Data search strategy. (PDF 29 kb)

Additional file 3: Data extraction spreadsheet. (PDF 41 kb)

Additional file 4: PRISMA checklist. (PDF 54 kb)

## References

[1] Ahmad SS, Amin TN, Ustianowski A (2016). Zika virus: management of infection and risk. BMJ (Clinical research ed).

[2] Boeuf P, Drummer HE, Richards JS, Scoullar MJ, Beeson JG (2016). The global threat of Zika virus to pregnancy: epidemiology, clinical perspectives, mechanisms, and impact. BMC Med.

[3] Brown C (2016). Zika virus outbreaks in Asia and South America. CMAJ.

[4] Petersen LR, Jamieson DJ, Powers AM, Honein MA, Virus Z (2016). N Engl J Med.

[5] Paixao ES, Barreto F, Teixeira Mda G, Costa Mda C, Rodrigues LC (2016). History, epidemiology, and clinical manifestations of Zika: a systematic review. Am J Public Health.

[6] Plourde AR, Bloch EM (2016). A literature review of Zika virus. Emerg Infect Dis.

[7] PAHO/WHO (2016). Guideline for Zika virus disease and complications surveillance.

[8] WHO (2016). Zika virus infection: global update on epidemiology and potentially associated clinical manifestations. Releve epidemiologique hebdomadaire.

[9] Singh RK, Dhama K, Malik YS, Ramakrishnan MA, Karthik K, Tiwari R, Saurabh S, Sachan S, Joshi SK (2016). Zika virus - emergence, evolution, pathology, diagnosis, and control: current global scenario and future perspectives - a comprehensive review. Vet Q.

[10] Posen HJ, Keystone JS, Gubbay JB, Morris SK. Epidemiology of Zika virus, 1947–2007. BMJ Global Health. 2016;1(2) 10.1136/bmjgh-2016-000087 PMC5321352 28588942

[11] WHO: Assessment and management of Guillain-Barré syndrome in the context of Zika virus infection. In: Interim Guidance update. Edited by Who/ZIKV. Geneva, Switzertland WHO; 2016: 11.

[12] Broutet N, Krauer F, Riesen M, Khalakdina A, Almiron M, Aldighieri S, Espinal M, Low N, Dye C (2016). Zika virus as a cause of neurologic disorders. N Engl J Med.

[13] Alvarado MG, Schwartz DA (2016). Zika Virus Infection in Pregnancy, Microcephaly, and Maternal and Fetal Health: What We Think, What We Know, and What We Think We Know. Arch Pathol Lab Med.

[14] Cugola FR, Fernandes IR, Russo FB, Freitas BC, Dias JL, Guimaraes KP, Benazzato C, Almeida N, Pignatari GC, Romero S (2016). The Brazilian Zika virus strain causes birth defects in experimental models. Nature.

[15] Faria NR, Azevedo Rdo S, Kraemer MU, Souza R, Cunha MS, Hill SC, Theze J, Bonsall MB, Bowden TA, Rissanen I (2016). Zika virus in the Americas: early epidemiological and genetic findings. Science (New York, NY).

[16] Moura da Silva AA, Ganz JS, Sousa PD, Doriqui MJ, Ribeiro MR, Branco MD, Queiroz RC, Pacheco MJ, Vieira da Costa FR, Silva FS (2016). Early growth and neurologic outcomes of infants with probable congenital Zika virus syndrome. Emerg Infect Dis.

[17] Panchaud A, Stojanov M, Ammerdorffer A, Vouga M, Baud D (2016). Emerging role of Zika virus in adverse fetal and neonatal outcomes. Clin Microbiol Rev.

[18] Adibi JJ, Marques ET, Cartus A, Beigi RH (2016). Teratogenic effects of the Zika virus and the role of the placenta. Lancet (London, England).

[19] Brasil P, Pereira JPJ, Moreira ME, Ribeiro Nogueira RM, Damasceno L, Wakimoto M, Rabello RS, Valderramos SG, Halai U-A, Salles TS (2016). Zika virus infection in pregnant women in Rio de Janeiro. N Engl J Med.

[20] Besnard M, Eyrolle-Guignot D, Guillemette-Artur P, Lastere S, Bost-Bezeaud F, Marcelis L, Abadie V, Garel C, Moutard ML, Jouannic JM, et al. Congenital cerebral malformations and dysfunction in fetuses and newborns following the 2013 to 2014 Zika virus epidemic in French Polynesia. Euro Surveill. 2016;21(13):​30181. 10.2807/1560-7917.ES.2016.21.13.30181 27063794

[21] Butler D (2016). First Zika-linked birth defects detected in Colombia. Nature.

[22] Ellington SR, Devine O, Bertolli J, Martinez Quinones A, Shapiro-Mendoza CK, Perez-Padilla J, Rivera-Garcia B, Simeone RM, Jamieson DJ, Valencia-Prado M (2016). Estimating the number of pregnant women infected with Zika virus and expected infants with microcephaly following the Zika virus outbreak in Puerto Rico, 2016. JAMA Pediatr.

[23] Franca GV, Schuler-Faccini L, Oliveira WK, Henriques CM, Carmo EH, Pedi VD, Nunes ML, Castro MC, Serruya S, Silveira MF (2016). Congenital Zika virus syndrome in Brazil: a case series of the first 1501 livebirths with complete investigation. Lancet (London, England).

[24] Russell K, Oliver SE, Lewis L, Barfield WD, Cragan J, Meaney-Delman D, Staples JE, Fischer M, Peacock G, Oduyebo T (2016). Update: interim guidance for the evaluation and Management of Infants with possible congenital Zika virus infection - United States, august 2016. MMWR Morb Mortal Wkly Rep.

[25] Karwowski MP, Nelson JM, Staples JE, Fischer M, Fleming-Dutra KE, Villanueva J, Powers AM, Mead P, Honein MA, Moore CA, et al. Zika virus disease: a CDC update for pediatric health care providers. Pediatrics. 2016;137(5):​e20160621. 10.1542/peds.2016-0621 27009036

[26] Reefhuis J, Gilboa SM, Johansson MA, Valencia D, Simeone RM, Hills SL, Polen K, Jamieson DJ, Petersen LR, Honein MA (2016). Projecting month of birth for at-risk infants after Zika virus disease outbreaks. Emerg Infect Dis.

[27] Jouannic JM, Friszer S, Leparc-Goffart I, Garel C, Eyrolle-Guignot D (2016). Zika virus infection in French Polynesia. Lancet (London, England).

[28] WHO (2016). Statement on the first meeting of the international health regulations (2005) (IHR 2005) emergency committee on Zika virus and observed increase in neurological disorders and neonatal malformations. Edited by statement W.

[29] Coyne CB, Lazear HM (2016). Zika virus - reigniting the TORCH. Nat Rev Micro.

[30] MINSALUD C (2016). Lineamientos para la provision efectiva de metodos de anticoncepcion a hombres y mujeres en edad fertil. dirigidos a POSTERGAR el embarazo en los 951 municipios por debajo de 2,200 msnm, en planes de contingencia para fiebre Zika. 00013–2016. Edited by Colombia MdSylPSd, vol. 00013–2016.

[31] de Carvalho BR, Taitson PF, Brandão KSAG, Ferriani RA, Nakagawa HM, Silva AA, JRC (2016). L: reproductive planning in times of Zika: getting pregnant or delaying plans? The opinion of the Brazilian Society of Assisted Reproduction Committee – a basis for a bioethical discussion. JBRA Assist Reprod.

[32] Ribeiro GS, Kitron U (2016). Zika virus pandemic: a human and public health crisis. Rev Soc Bras Med Trop.

[33] Pan American Health Organization., World Health Organization (2016). Zika suspected and confirmed cases reported by countries and territories in the Americas cumulative cases, 2015-2016. Edited by PAHO/WHO.

[34] Becker R (2016). Missing link: animal models to study whether Zika causes birth defects. Nat Med.

[35] Chowell G, Hincapie-Palacio D, Ospina J, Pell B, Tariq A, Dahal S, Moghadas S, Smirnova A, Simonsen L, Viboud C. Using phenomenological models to characterize transmissibility and forecast patterns and final burden of Zika epidemics. PLOS Currents Outbreaks. 2016;8:1–20. 10.1371/currents.outbreaks.f14b2217c902f453d9320a43a35b9583 PMC4922743 27366586

[36] Dyer O (2015). Zika virus spreads across Americas as concerns mount over birth defects. BMJ (Clinical research ed).

[37] Burke A, Moreau C (2016). Family planning and Zika virus: the power of prevention. Semin Reprod Med.

[38] McNeil DG, Symmes CJ. Colombia Is Hit Hard by Zika, but Not by Microcephaly. In: The New York Times. New York: The New York Times Company; 2016.

[39] Machado-Alba JE, Machado-Duque ME, Gaviria-Mendoza A, Orozco-Giraldo VA (2016). Hormonal contraceptive prescriptions in Colombia and Zika virus. Lancet (London, England).

[40] Pacheco O, Beltran M, Nelson CA, Valencia D, Tolosa N, Farr SL, Padilla AV, Tong VT, Cuevas EL, Espinosa-Bode A, et al. Zika virus disease in Colombia - preliminary report. N Engl J Med. 2016.​ 10.1056/NEJMoa1604037. 10.1056/NEJMoa1604037 27305043

[41] Camargo TM (2016). The debate on abortion and Zika: lessons from the AIDS epidemic. Cadernos de saude publica.

[42] Erdman JN (2012). Harm reduction, human rights, and access to information on safer abortion. Int J Gynecol Obstet.

[43] Erdman JN, DePiñeres T, Kismödi E (2013). Updated WHO guidance on safe abortion: health and human rights. Int J Gynecol Obstet.

[44] Carless W. A new bill aims to make Brazil's abortion law even tougher. In: Global Post. Part of a Series: Body Politics: The struggle for access to reproductive rights edn. Minneapolis: Public Radio Internationa (PRI) -Global Post; 2016.

[45] Diniz D (2016). Zika virus, women and ethics. Dev World Bioeth.

[46] Joerg D (2016). Countering Zika Globally and in the United States: Women’s Right to Self-Determination Must Be Central. Guttmacher Policy Review.

[47] Gawron LM, Watson K (2017). Documenting moral agency: a qualitative analysis of abortion decision making for fetal indications. Contraception.

[48] Guttmacher Institute. Abortion in Latin America and the Caribbean. In: Good reproductive health policy starts with credible research. vol. 2017. New York, USA: www.guttmacher.org; 2016. Fact sheet: Abortion in Latin America and the Caribbean [https://www.guttmacher.org/sites/default/files/factsheet/ib_aww-latin-america.pdf].

[49] Nolan R (2016). Innocents: Where pregnant women have more to fear than Zika. Harper’s.

[50] Mayor S (2016). Abortion requests increase in Latin America after Zika warning, figures show. BMJ (Clinical research ed).

[51] Aiken AR, Scott JG, Gomperts R, Trussell J, Worrell M, Aiken CE (2016). Requests for abortion in Latin America related to concern about Zika virus exposure. N Engl J Med.

[52] Collucci C (2016). Brazilian attorneys demand abortion rights for women infected with Zika. BMJ (Clinical research ed).

[53] Howard AR (2016). From Rubella to Zika: NEW LESSONS FROM AN OLD EPIDEMIC. Commonweal.

[54] Sedacca N (2016). Abortione in Latin America in international perspective: limitations and potentials of the use of human rights law to challenge restrictions. Dickson poon transnational law institute, School of law.

[55] Arksey H, O'Malley L (2005). Scoping studies: towards a methodological framework. Int J Soc Res Methodol.

[56] Davis K, Drey N, Gould D (2009). What are scoping studies? A review of the nursing literature. Int J Nurs Stud.

[57] Levac D, Colquhoun H, O’Brien KK (2010). Scoping studies: advancing the methodology. Implement Sci.

[58] Pluye P (2010). Collaborative development of a mixed methods appraisal tool: a public WIKI workspace. Edited by Department of Family Medicine MU, Montreal, Canada.

[59] Hodge JG, Corbett A, Repka A, Judd PJ (2016). Zika virus and global implications for reproductive health reforms. Disaster Med Public Health Prep.

[60] Bueno MAS, Grunspun H (2016). Bioethical considerations at times of Zika virus. Einstein (São Paulo).

[61] Galli B. Where is the right to abortion? Comment on the documentary Zika, the film. Cadernos de saude publica. 2016;32(6) 10.1590/0102-311XES010616 27383459

[62] Garsd J. Zika Virus Isn't The First Disease To Spark A Debate About Abortion. In: https://www.nprorg/. Washington, D.C.: National Public Radio, Inc. (NPR); 2016. [http://www.npr.org/sections/goatsandsoda/2016/01/31/464750384/Zika-virus-isnt-the-first-disease-to-spark-a-debate-about-abortion].

[63] Miller M (2016). With abortion banned in Zika countries, women beg on web for abortion pills. The Washington Post.

[64] Brosco JH, Brosco JP (2016). Zika as a catalyst for social change. Pediatrics.

[65] Stern AM (2016). Zika and reproductive justice. Cadernos de saude publica.

[66] Davies SE, Bennett B (2016). A gendered human rights analysis of Ebola and Zika: locating gender in global health emergencies. Int Aff.

[67] Harris LH, Silverman NS, Marshall MF (2016). The paradigm of the paradox: women, pregnant women, and the unequal burdens of the Zika virus pandemic. Am J Bioeth.

[68] Villa R (2016). Zika, or the burden of uncertainty. Clin Ter.

[69] Miller M (2016). Infected with dogma: how South America's response to the Zika virus fails women. Humanist.

[70] Young-Lee P. The three-letter word missing from the Zika warnings – men. The Guardian; 2016.

[71] Castro MC (2016). Zika virus and health Systems in Brazil: from unknown to a menace. Health Syst Reform.

[72] Goldthwaite LM, Velasquez G (2016). Family planning and the Zika era. Curr Opin Obstet Gynecol.

[73] Tavares MP, Foster AM (2016). Emergency contraception in a public health emergency: exploring pharmacy availability in Brazil. Contraception.

[74] Gostin LO, Hodge JG (2016). Zika virus and global health security. Lancet Infect Dis.

[75] Fernandez Anderson C (2016). Reproductive inequalities. NACLA Rep Am.

[76] Buchbinder M, Lassiter D, Mercier R, Bryant A, Lyerly AD (2016). Reframing conscientious care: providing abortion care when law and conscience collide. Hastings Cent Rep.

[77] Swanson KW (2015). The Doctor’s Dilemma: Paternalisms in the Medicolegal History of Assisted Reproduction and Abortion. J Law Med Ethics.

[78] Ballantyne A, Newson A, Luna F, Ashcroft R (2009). Prenatal diagnosis and abortion for congenital abnormalities: is it ethical to provide one without the other?. Am J Bioeth.

[79] O'Brien J (2016). Pope Francis should lift abortion bans to fight Zika. Conscience.

[80] Faúndes A, Távara L, Brache V, Alvarez F (2007). Emergency contraception under attack in Latin America: response of the medical establishment and civil society. Reprod Health Matters.

[81] Morgan LM, Roberts EFS (2012). Reproductive governance in Latin America. Anthropol Med.

[82] Pew Research Center. Numbers, facts and trends shaping your world. Chapter: social attitudes (views on abortion). In: Religion and public life. Vol. religion in Latin America. Washington D.C: PewResearchCenter. p. 2014.

[83] Perry CN, Beca IJ (2017). Virus Zika y Aborto por Correspondencia, Una Realidad Cercana A Chile. Rev Chilena Obstet Ginecol.

[84] Vlassoff M, Walker D, Shearer J, Newlands D, Singh S (2009). Estimates of health care system costs of unsafe abortion in Africa and Latin America. Int Perspect Sex Reprod Health.

[85] Prada E, Maddow-Zimet I, Juarez F (2013). The cost of Postabortion care and legal abortion in Colombia. Int Perspect Sex Reprod Health.

[86] Ventura M, de Camargo TMCR (2016). Direitos Reprodutivos e o Aborto: as mulheres na epidemia de Zika./reproductive rights and abortion: women in the Zika epidemic. Revista Direito e Práxis.

[87] Young-Lee P (2016). The three-letter word missing from the Zika warnings – men. The Guardian.

[88] Darney BG, Aiken ARA, Küng S (2017). Access to contraception in the context of Zika: health system challenges and responses. Obstet Gynecol.

