# Supplementary material for: The Zika epidemic and abortion in Latin America: a scoping review
Source: Glob Health Res Policy. 2018 May 3;3:15. doi: 10.1186/s41256-018-0069-8 (PMC5932843; doi:10.1186/s41256-018-0069-8)
Supplement: Supplementary file 1 — Description of country-specific information on cumulative Zika cases, CZS cases, abortion regulations, and abortion demand in 13 Latin American countries. (PDF 30 kb) [file 41256_2018_69_MOESM1_ESM.pdf]

Appendix 1

The zika epidemic and abortion in Latin America: a scoping review

Summary of current abortion policies, zika/CZS cases, ZIKV-related public health advisories, and demand for abortion pills such as mifepristone and misoprostol (which can be covertly requested via telemedicine by women in restrictive settings) during the last year in the Latin American countries with the highest ZIKV incidence (Aiken et al., 2016; Diniz, 2016; PAHO/WHO, 2016; Carless, 2016; Guttmacher Institute, 2016)

Table A.1. Description of country-specific information on cumulative zika cases, CZS cases, abortion regulations, and abortion demand in 13 Latin American countries.

Country	Abortion law	Legal grounds for abortion				Zika monitoring			Increased abortion pills requests [b]
		Women's life	Incest-Rape	Fetal anomalies	Socio-economic	All cases[a]	CZS cases	Pregnancy advisory	
**Brazil**	Illegal with some exceptions	Yes	Yes	No[c]	No	130,840	2,386	Yes	Yes
**Colombia**	Illegal with some exceptions	Yes	Yes	Yes	No	9,799	127	Yes	Yes
**Dominican Republic**	Illegal	No	No	No	No	345	59	No	No
**Guatemala**	Illegal with some exceptions	Yes	No	No	No	890	37	No	Yes
**Bolivia**	Illegal with some exceptions	Yes	Yes	No	No	192	14	No	Yes

**Puerto Rico**	Legal (with some restrictions)	Yes	Yes	Yes	Yes	38,940	12	Yes	ND[d]
**Panama**	Illegal with some exceptions	Yes	Yes	Yes	No	897	5	No	Yes
**El Salvador**	Illegal	No	No	No	No	51	4	Yes	Yes
**Nicaragua**	Illegal	No	No	No	No	2,060	2	No	Yes
**Argentina**	Illegal with some exceptions	Yes	Yes	No	No	26	2	No	Yes
**Paraguay**	Illegal with some exceptions	Yes	Yes	No	No	14	2	No	Yes
**Cuba**	Legal	Yes	Yes	Yes	Yes	187	0	No	ND
**Honduras**	Illegal with some exceptions	Yes	No	No	No	298	2	Yes	Yes

[a] Cumulative confirmed cases reported to PAHO by March 2, 2017 (http://www.paho.org/hq/index.php?option=com_docman&task=doc_view&Itemid=270&gid=38464&lang=es);

[b] Higher than expected Women on Web (https://www.womenonweb.org/) abortion pills requests following the PAHO announcement

[c] Only anencephaly;

[d] ND= No Data;

Data source: (Aiken et al., 2016; Diniz, 2016; PAHO/WHO, 2016; Carless, 2016; Guttmacher Institute, 2016).
