# Supplementary material for: The Zika epidemic and abortion in Latin America: a scoping review
Source: Glob Health Res Policy. 2018 May 3;3:15. doi: 10.1186/s41256-018-0069-8 (PMC5932843; doi:10.1186/s41256-018-0069-8)
Supplement: Supplementary file 2 — Data search strategy. (PDF 29 kb) [file 41256_2018_69_MOESM2_ESM.pdf]

## The zika epidemic and abortion in Latin America: a scoping review

## Appendix 2. Data search strategy:

Search 1:
Search **(zika[MeSH Terms]) AND abortion[MeSH Terms]**

Search 2:
Search **("congenital zika syndrome") AND "abortion"**

Search **(zika birth defects) AND abortion**

Search 3:
Search **(zika) AND antenatal screening**

Search 4:
Search **(zika birth defects) AND bioethic**

Search 5:
Search **(zika birth defects) AND "public health program"**

Search 6:
Search **((zika) AND antenatal screening) AND abortion**

Search 7:
Search **(abortion) AND zika**

Search 8:
Search **(((((("abortion") AND "zika") OR "zika disease") OR "zika virus infection") OR "zika infection") OR "zika syndrome") OR "zika virus"**

Search 9:
Search **((("microcephaly") AND zika) AND "antenatal care") OR "antenatal screening"**

Search 10:
Search **((("microcephaly") AND "zika")) AND antenatal care**

Search 11:
Search **((("microcephaly") AND "zika")) AND antenatal screening**

Search 12:
Search **(((((("microcephaly") AND "zika")) AND antenatal screening)) OR (((("microcephaly") AND "zika")) AND antenatal care)) AND "abortion"**

Search 13:
Search **(((Search AND (((((("abortion") AND "zika") OR "zika disease") OR "zika virus infection") OR "zika infection") OR "zika syndrome") OR "zika virus") AND ("2015/01/01"[PDat] : "2017/12/31"[PDat]))) AND "congenital zika syndrome" AND (("2015/01/01"[PDat] : "2017/12/31"[PDat]))**

**Search in Google scholar**

zika abortion latin america -animal -mice -mouse –rhesus (2015-2017)
