# Supplementary material for: The Zika epidemic and abortion in Latin America: a scoping review
Source: Glob Health Res Policy. 2018 May 3;3:15. doi: 10.1186/s41256-018-0069-8 (PMC5932843; doi:10.1186/s41256-018-0069-8)
Supplement: Supplementary file 3 — Data extraction spreadsheet. (PDF 41 kb) [file 41256_2018_69_MOESM3_ESM.pdf]

## The zika epidemic and abortion in Latin America: a scoping review

### Appendix 3. Documents reviewed:

| AUTHOR | TITLE | JOURNAL | PUBYEAR | TYPE_DOC |
|---|---|---|---|---|
| **Aiken, A. R.; Scott, J. G.; Gomperts, R.; Trussell, J.; Worrell, M.; Aiken, C. E.** | Requests for Abortion in Latin America Related to Concern about Zika Virus Exposure | The New England journal of medicine; N Engl J Med | 2016 | QT/MM |
| **Baum, Paige; Fiastro, Anna; Kunselman, Shane; Vega, Camila; Ricardo, Christine; Galli, Beatriz; Nascimento, Marcos** | Garantindo uma resposta do setor de saúde com foco nos direitos das mulheres afetadas pelo vírus Zika | Cadernos de Saúde Pública | 2016 | QL |
| **Brosco, J. H.; Brosco, J. P.** | Zika as a Catalyst for Social Change | PEDIATRICS PEDIATRICS | 2016 | QL |
| **Bueno, Marco Aurélio Scarpinella.; Grunspun, Henrique** | Bioethical considerations at times of Zika virus | Einstein (São Paulo) | 2016 | QL |
| **Burke, A.; Moreau, C.** | Family Planning and Zika Virus: The Power of Prevention | Seminars in reproductive medicine; Semin Reprod Med | 2016 | QT |
| **Camargo, T. M.** | The debate on abortion and Zika: lessons from the AIDS epidemic | Cadernos de saude publica; Cad Saude Publica | 2016 | QL |
| **Carvalho, Marilia Sá** | Zika in Cadernos de Saúde Pública/Reports in Public Health: again? | Cadernos de saude publica | 2016 | QL |
| **Castro, Marcia C.** | Zika Virus and Health Systems in Brazil: From Unknown to a Menace | Health Systems & Reform | 2016 | QL |
| **Collucci, C.** | Brazilian attorneys demand abortion rights for women infected with Zika | BMJ (Clinical research ed.); Bmj | 2016 | QL |
| **Davies, Sara E.; Bennett, Belinda** | A gendered human rights analysis of Ebola and Zika: locating gender in global health emergencies | International Affairs | 2016 | QL |
| **de Campos, Thana Cristina** | Zika, public health, and the distraction of abortion | Medicine, Health Care and Philosophy | 2016 | QL |
| **Diniz, Debora** | Zika virus, women and ethics | Developing World Bioethics | 2016 | QL |

| | | | | |
|---|---|---|---|---|
| **Diniz, Debora; Gumieri, Sinara; Bevilacqua, Beatriz Galli; Cook, Rebecca J.; Dickens, Bernard M.** | Zika virus infection in Brazil and human rights obligations | International Journal of Gynecology & Obstetrics | 2017 | QL |
| **Galli, B.** | Where is the right to abortion? Comment on the documentary Zika, The Film | Cadernos de saude publica; Cad Saude Publica | 2016 | QL |
| **Garsd, J.** | Zika Virus Isn't The First Disease To Spark A Debate About Abortion | | 2016 | QL |
| **Goldthwaite, L. M.; Velasquez, G.** | Family planning and the Zika era | Current opinion in obstetrics & gynecology; Curr Opin Obstet Gynecol | 2016 | QL |
| **González-Vélez, Ana Cristina** | Comment on the article by Baum et al. | Cadernos de Saúde Pública | 2016 | QL |
| **Gostin, L. O.; Hodge, J. G., Jr.** | Zika virus and global health security | Lancet Infect Dis | 2016 | QL |
| **Harris, Lisa H.; Silverman, Neil S.; Marshall, Mary Faith** | The Paradigm of the Paradox: Women, Pregnant Women, and the Unequal Burdens of the Zika Virus Pandemic | The American Journal of Bioethics | 2016 | QL |
| **Hodge, J. G.; Corbett, A.; Repka, A.; Judd, P. J.** | Zika Virus and Global Implications for Reproductive Health Reforms | Disaster Med Public Health Prep | 2016 | QL |
| **Howard, Agnes R.** | From Rubella to Zika: NEW LESSONS FROM AN OLD EPIDEMIC | Commonweal | 2016 | QL |
| **Leite, Marianna** | The Outbreak of the Zika Virus and Reproductive Rights in Latin America | E-International Relations | 2016 | QL |
| **Mayor, S.** | Abortion requests increase in Latin America after Zika warning, figures show | BMJ (Clinical research ed.); Bmj | 2016 | QL |
| **Miller, Merrill** | Infected with Dogma: How South America's Response to the Zika Virus Fails Women | Humanist | 2016 | QL |
| **Miller M, .** | With abortion banned in Zika countries, women beg on web for abortion pills | The Washington Post | 2016 | QL |
| **Mohapatra, Seema** | Law in the Time of Zika | SSRN | 2016 | QL |
| **Nolan, Rachel.** | Innocents: Where pregnant women have more to fear than Zika | Harper's | 2016 | QL |
| **Roa, M.** | Zika virus outbreak: reproductive health and rights in Latin America | Lancet (London, England); Lancet | 2016 | QL |

| | | | | |
|---|---|---|---|---|
| **Schwartz, Madeleine** | What Happens When Zika Hits the Country With the World's Strictest Abortion Laws? | The Nation | 2016 | QL |
| **Sedacca, Natalie** | Abortion in Latin America in International Perspective: Limitations and Potentials of the Use of Human Rights Law to Challenge Restrictions | Dickson Poon Transnational Law Institute, School of Law | 2016 | QL |
| **Sriwijitalai, Won; Wiwanitkit, Viroj** | Zika Virus Infection in Pregnant Women: Topic for Discussion | Revista Brasileira de Ginecologia e Obstetrícia/RBGO Gynecology and Obstetrics | 2016 | QL |
| **Stern, Alexandra Minna** | Zika and reproductive justice | Cadernos de Saúde Pública | 2016 | QL |
| **Velez, Ana Cristina Gonzalez; Diniz, Simone G.** | Inequality, Zika epidemics, and the lack of reproductive rights in Latin America | RHM Reproductive Health Matters | 2016 | QL |
| **Ventura, Miriam; de Camargo, Thais Medina Coeli Rochel** | Direitos Reprodutivos e o Aborto: As mulheres na epidemia de Zika./Reproductive rights and abortion: Women in the Zika epidemic | Revista Direito e Práxis | 2016 | QL |
| **Villa, R.** | Zika, or the burden of uncertainty | Clin Ter | 2016 | QL |
| **Young-Lee P.** | The three-letter word missing from the Zika warnings – men | The Guardian | 2016 | QL |

**TYPE_DOC** = Type of document: QT=Qualitative; QL= Quantitative, MM=Mixed Methods
